# Fellow-Workers and the Roll of Honour

**Published:** 1915-11-27

**Authors:** 


### ROUND THE WORLD.

[The Editor invites Early Information and Contributions Marked "Fellow-Workers."]

### Fellow-Workers and the Roll of Honour.

THE medical profession has gained yet another distinction in this campaign in the person of Temporary Lieutenant George Allan Maling, M.B., R.A.M.C., who for most conspicuous bravery and devotion to duty during the heavy fighting near Fauquissart on September 25 has been awarded the Victoria Cross.

LIEUTENANT MALING gained the M.B. degree of Oxford University last year, and it is less than a year since he became registered as a medical practitioner. It is recorded of this young man that he "worked incessantly with untiring energy from 6.15 A.M. on September 25 till 8 A.M. on the 26th collecting and treating in the open, under heavy shell fire, more than 300 men. At about 11 A.M. on the 25th he was flung down and temporarily stunned by the bursting of a large high-explosive shell, which wounded his only assistant and killed several of his patients. A second shell soon after covered him and his instruments with *débris*, but his courage and zeal never failed him, and he continued his gallant work single-handed."

FURTHER lists of awards for gallantry which have been published this week contain the names of two medical officers of the Royal Army Medical Corps—namely, Captain Maurice Holdsworth Barton, M.R.C.S., L.R.C.P., attached 5th Battalion of the Leicestershire Regiment, and Captain Samuel Russell Foster, M.B., 2nd North Midland Field Ambulance, R.A.M.C. (T.F.).

CAPTAIN BARTON became registered as a medical practitioner only nine months ago, but this is not the first time that his bravery and good work have been brought to notice. He has now been awarded the Military Cross for conspicuous gallantry and devotion to duty at Hohenzollern redoubt on October 13 in tending and bringing in wounded under fire. He also rallied and sent forward men who had become scattered.

CAPTAIN FOSTER has been awarded the Military Cross for gallantry at Hohenzollern redoubt on October 16. He went to the relief of an officer and some wounded men who were lying in a trench between the firing lines, passing over a considerable space of open ground in broad daylight under heavy shell fire, machine-gun and rifle fire. He spent eight hours in this trench tending severely wounded men. Captain Foster obtained his medical degree at Belfast three years ago.

WE are glad to see the announcement in *St. Mary's Hospital Gazette* that Colonel G. T. K. Maurice, C.M.G. (R.A.M.C.), who had the bad luck to break his leg in a riding accident last March while on active service, is quite fit again. He is at present commandant of the Tent Hospital at Malta—a hospital of some 3,000 beds—and was decorated last March for his services in command of No. 8 Field Ambulance.

SIR ST. CLAIR THOMSON, M.D., F.R.C.S., F.R.C.P., whom the King of the Belgians has appointed a Commander of the Order of Leopold, is the popular Professor of Laryngology and Physician for Diseases of the Throat and Nose in King's College Hospital. He holds many appointments, is corresponding member of many foreign Laryngological Societies, and European editor of the *Laryngoscope*.

CAPTAIN E. COOMBER HOBBS, R.A.M.C., who has been invalided home from the Dardanelles with dysentery, is another old St. Mary's man. Before his appointment to the Borough Mental Hospital, Leicester, he had held resident posts at Westminster Hospital, the London Lock Hospital, and the Miller Hospital, Greenwich.

MAJOR HERMAN STEDMAN, F.R.C.S. (Ed.), late R.A.M.C. (T.F.), who has been similarly honoured by the King of the Belgians, has devoted himself to gynæcology and abdominal injury since 1905. He was formerly attached to the 1st London Brigade R.F.A. For him is claimed the distinction of being the first medical man to use a motor-car for professional service in the United Kingdom.

ST. MARY'S HOSPITAL has suffered a severe loss by the death of Mr. George Coats, M.D., F.R.C.S., assistant ophthalmic surgeon to the hospital, who died early this month. He was elected to the staff in 1911, when he already occupied a very distinguished position in ophthalmological circles. As the *Gazette* observes, "It is given to few men to achieve a European reputation at so early an age. His industry was only equalled by the excellence of his work." It is with extreme regret that we record the close of a brilliant career.

DR. Major Greenwood has been appointed to the office of Deputy Coroner for the districts of Shoreditch, Bethnal Green, and Hackney. This gentleman, who is a well-known practitioner in the Hackney Road, is a barrister-at-law of Lincoln's Inn, and also holds the degree of LL.B. of the University of London. He is thus doubly qualified for the important office of Deputy Coroner for such a large and populous district. Dr. Major Greenwood qualified from the London Hospital in 1876 as a Member of the Royal College of Surgeons, England. He holds also the M.D. degree of Brussels and a diploma in public health. Several public appointments in his district are held by him, and he has a large general practice. On the Poor Law Dr. Greenwood is an authority. He is the editor of the *Poor-Law Medical Officer* and honorary secretary to the Poor-Law Medical Officers' Association.

DR. BARCLAY S. BARON, the newly-elected Lord Mayor of Bristol, is an Edinburgh University graduate, and is consulting physician for diseases of the throat and nose at Bristol General Hospital. He is an ex-president of the Bristol Laryngological, Rhinological, and Otological Association. He has contributed to medical literature various papers on these specialties. Dr. R. J. Smith, the newly-elected Lord Mayor of Cardiff, is Chairman of the Cardiff Division of the British Medical Association, and is a graduate of the Royal University of Ireland.

COL. SIR W. B. LEISHMAN, C.B., F.R.S., who opened the adjourned debate on paratyphoid fever at the Royal Society of Medicine on November 23, is adviser in pathology to the British Expeditionary Force. He was Professor of Pathology at the Army Medical College from 1903 to 1913, and is one of the foremost scientific bacteriologists of the day. His reputation was originally laid in India, and his name is familiar to students of medicine as the deviser of Leishman's stain for bacteria, as well as in connection with the Leishman-Donovan micro-organisms.

